# Therapeutic effects of mesenchymal stromal cells transplantation in animal models of chronic obstructive pulmonary disease: a systematic review and meta-analysis of emphysema and lung inflammation models

**DOI:** 10.3389/fcell.2025.1739905

**Published:** 2026-01-08

**Authors:** Yuan Zhao, Shuhui Zhou, Juan Pei, Ying Meng, Zhengyi Zhang

**Affiliations:** 1 General Medical Department, Lanzhou University Second Hospital, Lanzhou, China; 2 Lanzhou University, Lanzhou, China; 3 Lanzhou University Second Hospital, Lanzhou, China

**Keywords:** animal models, chronic obstructive pulmonary disease, mesenchymal stromal cells, meta-analysis, transplantation strategies

## Abstract

**Objective:**

Chronic obstructive pulmonary disease (COPD) presents core pathological changes that current medications cannot reverse. Mesenchymal stromal cell (MSC) transplantation has shown therapeutic potential in preclinical studies; however, significant heterogeneity and inconsistency exist in animal experiments simulating key COPD pathologies (such as emphysema and inflammation) based on acute injury models. We aim to systematically evaluate the efficacy of MSC transplantation in animal models simulating COPD pathology through a meta-analysis and to explore the impact of key strategies such as administration routes and dosages on efficacy.

**Methods:**

A systematic search was performed in PubMed, Web of Science, Embase, and Scopus databases (up to 1 July 2025) to identify randomized controlled trials (RCTs) involving MSC transplantation in animal models of simulated COPD pathology. Risk of bias was assessed using the SYRCLE tool, and meta-analysis was conducted using R software.

**Results:**

A total of 40 studies were included. The meta-analysis revealed that MSC transplantation significantly improved alveolar structural damage compared to control groups (MLI: SMD = −2.84, 95% CI: 3.22 to −2.45), increased anti-inflammatory IL-10 levels (SMD = 6.54, 95% CI: 2.08–11.00), reduced pro-inflammatory TNF-α levels (SMD = −1.61, 95% CI: 2.72 to −0.5), and significantly inhibited pulmonary tissue cell apoptosis (SMD = −4.06, 95% CI: 5.71 to −2.41). Subgroup analysis showed that intratracheal transplantation was more effective than intravenous transplantation in improving MLI, enhancing IL-10 levels, and reducing apoptosis. Moreover, the therapeutic effects were dose-dependent, with higher doses (≥5 × 10^6^) generally yielding superior outcomes. Publication bias assessment for MLI suggested potential bias; however, the adjusted combined effect size remained statistically significant, confirming the robustness of the conclusion that MSCs significantly improve alveolar structure.

**Conclusion:**

MSC transplantation exerts multiple therapeutic effects by alleviating emphysema, regulating inflammatory balance, and inhibiting cell apoptosis. The study further identifies intratracheal delivery and higher cell dosages as promising optimization strategies for MSC transplantation. These findings provide critical references for the standardized design of future preclinical studies and the selection of parameters for subsequent clinical trials, while the differences in disease progression between animal models and human conditions remain key factors to consider for future clinical translation.

## 1 Introduction

Chronic obstructive pulmonary disease (COPD) is a progressive inflammatory disorder characterized by persistent airflow limitation, affecting over 213 million people globally. The prevalence is expected to rise to 600 million by 2050, with more than 5.4 million deaths annually, making it the third leading cause of death worldwide (Christenson et al., 2022; Celli et al., 2022; Agustí et al., 2023; Wang Z. et al., 2025; Boers et al., 2023). The high prevalence, disability rate, and mortality associated with COPD present significant public health challenges. Although current pharmacological treatments, such as bronchodilators and inhaled corticosteroids, has been shown to alleviate clinical symptoms and slow disease progression to some extent, they cannot reverse the core pathological changes, such as airway remodeling and lung parenchymal destruction (Christenson et al., 2022; Agustí et al., 2023; Kapellos et al., 2023). Therefore, there is an urgent need for innovative therapeutic strategies that promote lung tissue regeneration and repair.

In recent years, mesenchymal stromal cell (MSC) have shown substantial promise in regenerative medicine due to their immunomodulatory, anti-inflammatory, and potent paracrine effects (Saba et al., 2021). MSCs have been widely studied for their therapeutic potential in respiratory diseases, including COPD, asthma, and idiopathic pulmonary fibrosis, positioning them as strong candidates for lung injury repair. Numerous preclinical studies indicate that MSC transplantation can alleviate lung inflammation, inhibit alveolar structural damage, and promote tissue repair in animal models simulating COPD pathology, offering hope for reversing lung damage (Abbaszadeh et al., 2022; Lai and Guo, 2024). However, existing animal experimental evidence is characterized by significant heterogeneity, with variations in key parameters such as MSC transplantation dose, administration routes, and outcome measures, leading to inconsistent conclusions and difficulty in standardizing efficacy evaluations. This variability has hindered the translation of the most promising therapeutic strategies into clinical trials (Lai and Guo, 2024; Chen et al., 2021). Furthermore, while Liu et al. provided a preliminary summary of preclinical studies on MSC therapy for COPD, their early meta-analysis has notable limitations (Liu et al., 2016). It lacks subgroup analyses focused on critical strategies such as transplantation dosage and administration routes, has a limited range of outcome measures, and has not incorporated the substantial new research evidence that has emerged since its publication in 2016. These factors render Liu et al.'s conclusions inadequate for guiding the selection of optimal transplantation strategies and fail to reflect the latest advancements in the field. Given this, the aim of this study is to systematically evaluate the overall therapeutic efficacy of MSCs in animal models simulating COPD pathology, exploring their effects under various conditions, and filling the critical gap in evidence from basic research to clinical translation.

## 2 Methods

### 2.1 Inclusion and exclusion criteria

Studies were eligible for inclusion if they were randomized controlled trials (RCTs) involving COPD animal models established by well-recognized methods, such as elastase instillation or chronic cigarette smoke exposure, regardless of animal species. It is important to note that there is currently no ideal animal model that perfectly replicates the complex, chronic, and progressive course of human COPD. The models included in this study, such as elastase instillation and cigarette smoke exposure, simulate the core pathological features of human COPD, namely, emphysema (alveolar structural damage) and chronic lung inflammation, through acute or subacute injury. These models provide a controlled and reliable reference for investigating the efficacy of MSC interventions on these specific pathological processes; however, the differences in pathophysiology and irreversibility between these models and human disease must be considered as critical factors when interpreting the results of this study and translating them to clinical applications.

Interventions included various MSC types, such as adipose-derived MSCs (ADMSC), bone marrow-derived MSCs (BMSC), and umbilical cord-derived MSCs (UCMSC). Control groups included positive controls (e.g., comparisons between different MSC types or different transplantation routes) and negative controls (e.g., placebo, phosphate-buffered saline, saline).

The primary outcome measures predefined in this study are: mean linear intercept (MLI), interleukin-10 (IL-10), tumor necrosis factor-alpha (TNF-α), and TUNEL-positive cell counts. The selection of these four primary outcome measures is based on their comprehensive coverage of the core pathological features of COPD: MLI serves as the gold standard morphological measure for assessing the degree of emphysema; TNF-α and IL-10 represent a pair of prototypical pro-inflammatory and anti-inflammatory factors used to evaluate the regulatory effects of MSCs on inflammatory imbalance; and TUNEL-positive cell counts directly reflect levels of apoptosis, a process that links chronic inflammation to lung structural damage.

Exclusion criteria included: 1) studies that did not explicitly report the specific transplantation route and cell dose of MSCs; 2) studies that did not provide extractable data for any of the four primary outcome measures; 3) non-English language publications; 4) studies where the full text could not be obtained, or where original data could not be acquired after contacting the authors; and 5) studies classified as reviews, meta-analyses, case reports, commentaries, letters, or conference abstracts.

### 2.2 Literature search strategy

A comprehensive electronic database search was conducted on 1 July 2025, across PubMed, Web of Science, Embase, and Scopus. The search strategy was based on a combination of controlled vocabulary terms and free-text terms, including keywords related to mesenchymal stromal cells, chronic obstructive pulmonary disease, and animal experiments. The search strategy was adapted for each database to align with their specific indexing rules, as detailed in Supplementary Table 1. To minimize publication bias and search omissions, we also performed supplementary manual searches, including reviewing the reference lists of relevant systematic reviews and meta-analyses. The full protocol for this study has been registered on the international prospective systematic review registry platform (PROSPERO).

### 2.3 Literature screening, data extraction, and risk of bias assessment

Literature screening and data extraction were independently performed by two researchers trained in systematic review methodology. Screening was initially based on titles and abstracts, with full-text articles obtained for further detailed evaluation. Any disagreements were resolved through discussion or by arbitration from a third senior researcher. Data extraction was carried out using a pre-designed and tested standardized electronic spreadsheet, which included the following information: 1) Study characteristics: first author, publication year, country, baseline characteristics of the animal models (species, sex, mean weight or age range); 2) Experimental design details: final sample size in both experimental and control groups, specific methods for modeling; 3) Intervention details: type of MSCs, tissue source, transplantation route, and MSC cell dose; 4) Control measures; 5) Endpoint values for all relevant outcome measures. Risk of bias in the included studies was assessed using the SYRCLE risk of bias tool, specifically designed for animal experiments (Hooijmans et al., 2014). The two researchers independently evaluated each study across 10 domains: sequence generation, baseline characteristics, allocation concealment, random housing, blinding of the study (performance bias), blinding of outcome assessment (detection bias), incomplete data, selective reporting, and other potential biases, in order to assess the overall methodological quality. It is noteworthy that for the criterion of “random housing,” a low risk of bias was determined only if the study explicitly stated that animals were randomly assigned to different cages during the intervention period to balance environmental factors. For “study blinding (performance bias),” a study was classified as low risk only if it met two conditions: it must clearly state that blinding was applied to personnel responsible for daily animal management and intervention implementation, and it must provide a detailed description of the specific blinding methods used (e.g., using identical-looking cell suspensions and placebos prepared by researchers not involved in group assignments). For “outcome assessment blinding (detection bias),” the sole criterion for low risk was whether the study explicitly stated that blinding was applied to personnel responsible for measuring final outcome indicators, such as data collection, histological analysis, or ELISA assays. If the original literature did not provide descriptions of these key pieces of information, the corresponding bias risk was classified as “unclear”.

### 2.4 Statistical analysis

All statistical analyses were performed using R statistical software (version 4.4.2), utilizing packages such as *meta*, *metafor*, *openxlsx*, *dplyr*, and *ggplot2*. For continuous outcome measures (MLI, IL-10, TNF-α, TUNEL-positive cell counts), standardized mean differences (SMD) and their 95% confidence intervals (95% CI) were calculated as the combined effect sizes. The choice of SMD was made due to the heterogeneity in measurement units and experimental conditions across the included studies. By eliminating differences in measurement scales, SMD allows for the combination and comparison of results from different studies, thereby providing a more robust estimate of the true treatment effect. Heterogeneity between studies was assessed using Cochran’s Q test (with a significance level set at α = 0.10) and quantified using the I^2^ statistic. If I^2^ ≤ 50%, heterogeneity was considered acceptable, and a fixed-effect model was used for the meta-analysis; if I^2^ > 50%, indicating substantial heterogeneity, a random-effects model was employed, and the restricted maximum likelihood method was used to estimate the between-study variance (τ^2^). To explore the potential sources of heterogeneity, predefined subgroup analyses based on key covariates, including MSC transplantation route and dose, were conducted. Publication bias was quantitatively assessed using Egger’s linear regression test and Begg’s rank correlation test, with visual assessment through funnel plots. If evidence of publication bias was identified, a trim-and-fill method was used to evaluate its impact on the overall results.

## 3 Results

### 3.1 Literature search results

**FIGURE 1:**
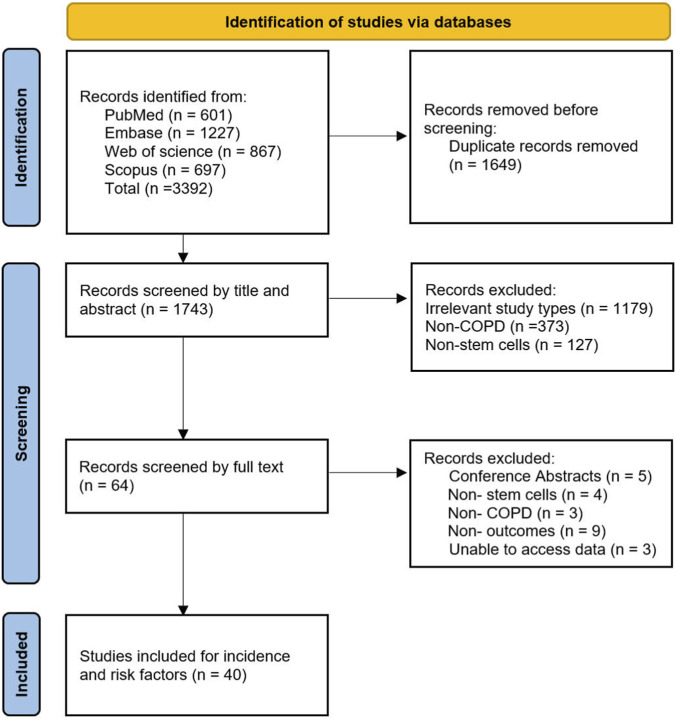
Flowchart of the literature screening process.

The initial search identified 3,392 potential studies related to MSC treatment in animal models simulating COPD pathology. After excluding 1,649 duplicate records, 1,743 articles were screened based on titles and abstracts, with 1,679 studies excluded for not meeting the inclusion criteria. Subsequently, 64 full-text articles were assessed, and a total of 40 studies were included in the final analysis. The detailed screening process is illustrated in Figure 1.

### 3.2 Basic characteristics of included studies

The 40 studies included in the final analysis were all randomized controlled animal trials published between 2008 and 2025. The animal species used included C57BL/6 mice (19 studies), SD rats (13 studies), Lewis rats (3 studies), BALB/c mice (1 study), ICR mice (1 study), NSG mice (1 study), SCID beige mice (1 study), and Wistar rats (1 study). Of these, 17 studies used male animals, 14 used female animals, and nine studies did not report the sex of the animals. The body weights of rats ranged from 170 to 360 g, and mice ranged from 18 to 25 g; however, 24 studies did not report body weight. The age range of the animals was between 3 and 12 weeks, with 14 studies not reporting the age of the animals. The sample sizes across the studies ranged from 10 to 60 animals, totaling 757 subjects.

The methods used to model COPD pathology in animals were diverse, primarily including cigarette smoke exposure, intratracheal injection of elastase, tracheal instillation of lipopolysaccharide, administration of the VEGF receptor blocker SU5416, ozone exposure, intratracheal injection of papain, cobalt-60 radiation, and infection with *Haemophilus* influenzae. The MSC types used predominantly included bone marrow-derived MSCs (BMSC) and human umbilical mesenchymal stromal cells (HUMSC), along with other sources such as human umbilical cord blood, human fallopian tube, amniotic fluid, and adipose-derived MSCs (ADMSC). The MSC transplantation routes included intratracheal injection (IT), intravenous injection (IV), and intraperitoneal injection (IP). The cell doses used in the transplantation varied widely, ranging from 0.01 × 10^6^ to 30 × 10^6^ cells. We categorized the doses into three levels: low dose (<1 × 10^6^), moderate dose (1 × 10^6^ to 5 × 10^6^), and high dose (≥5 × 10^6^) (Supplementary Table 2).

### 3.3 Risk of bias assessment

**FIGURE 2:**
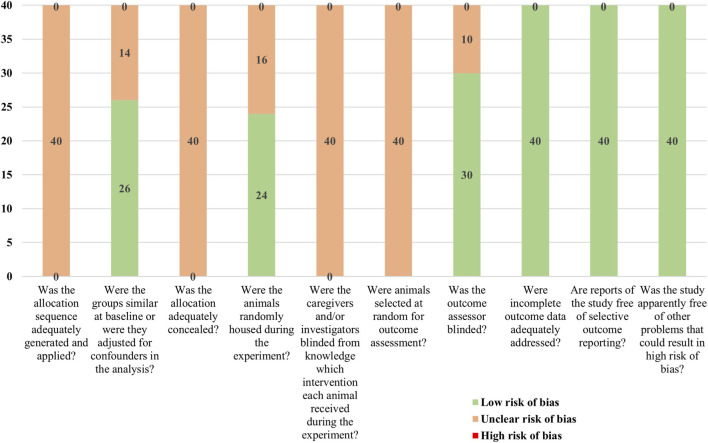
Risk of bias assessment results for included studies.

Although all included studies were reported as randomized controlled trials, none provided detailed information on the specific method of random sequence generation. Twenty-six studies reported comparability between the experimental and control groups in terms of baseline characteristics such as sex, weight, and age. None of the studies explicitly described whether allocation concealment was implemented or whether blinding was applied to the animal handlers or researchers. However, 24 studies mentioned random housing of animals during the experiment, and 30 studies confirmed that blinding was applied during the outcome assessment stage. All studies fully reported the predefined outcome measures, and there were no instances of animal dropouts. Overall, the included studies presented a high risk of bias in randomization methods and allocation concealment but showed a relatively low risk of bias in the blinding of outcome measurement and data completeness. Detailed risk of bias results are shown in Figure 2.

### 3.4 Meta-analysis results

#### 3.4.1 MLI

**FIGURE 3:**
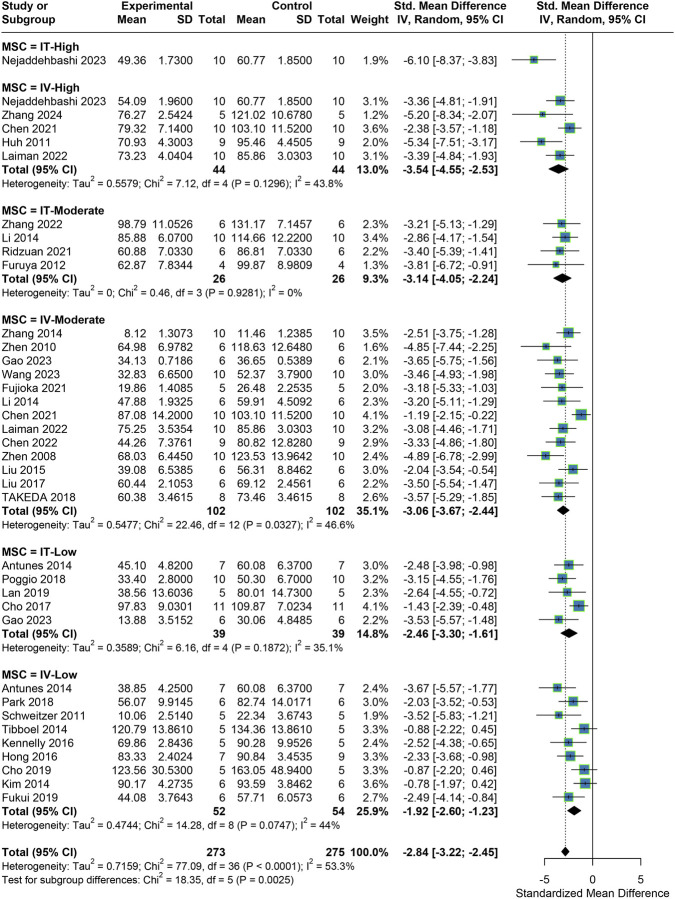
Meta-analysis results for MLI.

A total of 33 studies reported MLI, a key morphological indicator. The meta-analysis (fixed-effect model) revealed that MSC treatment significantly reduced MLI compared to the placebo control group, with a pooled effect size of SMD = −2.84 (95% CI: 3.22, −2.45). Given the moderate heterogeneity observed (I^2^ = 53.3%), subgroup analyses based on transplantation dose and route were performed. The subgroup analysis showed significant improvement in MLI across all dose and route categories, with heterogeneity reduced to low levels (I^2^ < 50%) within each subgroup. Additionally, the degree of MLI improvement increased with higher transplantation doses, and IT consistently outperformed IV at the same doses (Figure 3).

#### 3.4.2 IL-10

**FIGURE 4:**
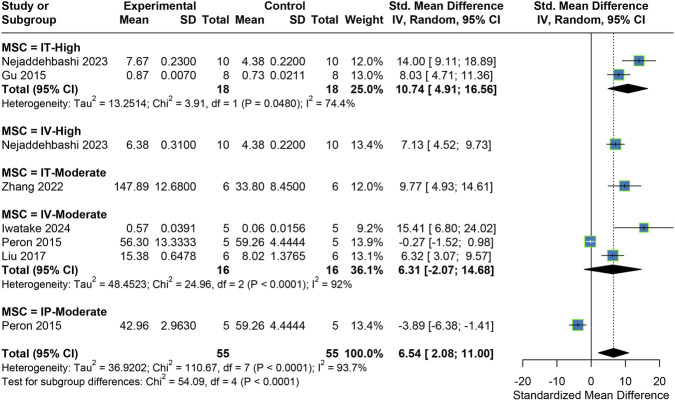
Meta-analysis results for IL-10.

Six studies measured the anti-inflammatory cytokine IL-10. The random-effects model meta-analysis revealed that MSC treatment significantly elevated IL-10 levels overall (SMD = 6.54, 95% CI: 2.08, 11.00). Subgroup analysis showed that moderate doses administered IP unexpectedly led to a decrease in IL-10 levels, while moderate doses administered IV had no significant effect. In contrast, moderate doses administered IT and high doses administered both IT and IV significantly increased IL-10 levels. Notably, at high doses, IT showed superior effects compared to IV (Figure 4).

#### 3.4.3 TNF-α

**FIGURE 5:**
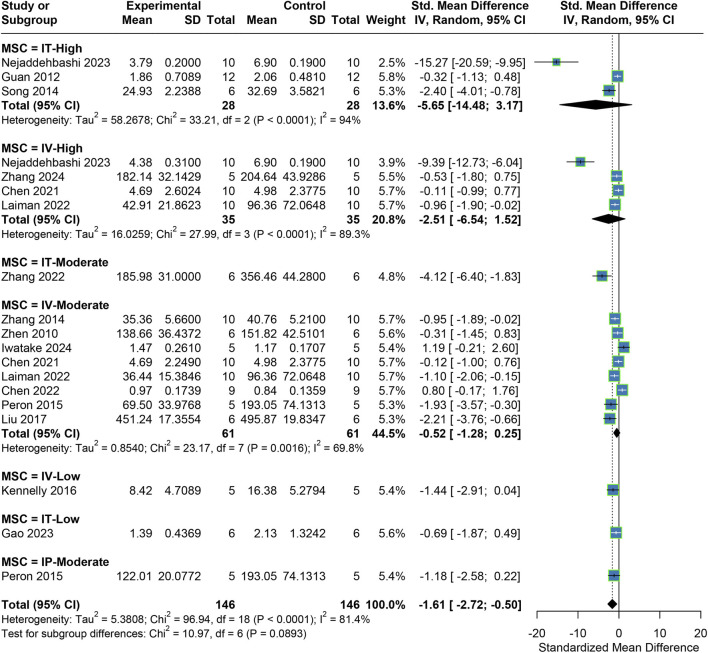
Meta-analysis results for TNF-α.

Fifteen studies reported TNF-α levels. The results of the meta-analysis using a random-effects model indicated that MSC treatment significantly reduced TNF-α levels compared to the placebo group (SMD = −1.61, 95% CI: 2.72 to −0.5). Further subgroup analysis revealed that only moderate doses administered via the intratracheal route were effective in significantly lowering TNF-α levels, while administration other routes and dosages did not achieve a reduction in TNF-α levels (Figure 5).

#### 3.4.4 Cell apoptosis

**FIGURE 6:**
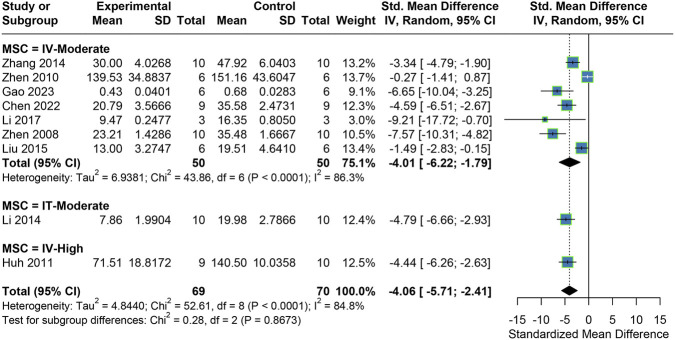
Meta-analysis results for apoptosis.

Nine studies evaluated cell apoptosis using TUNEL staining. The random-effects model meta-analysis revealed that MSC treatment significantly reduced apoptosis levels in animal models simulating COPD pathology (SMD = −4.06, 95% CI: 5.71, −2.41). Subgroup analyses, which included moderate-dose intratracheal injection and moderate and high-dose intravenous injection, showed that apoptosis inhibition increased with higher transplantation doses. Additionally, at the same dose, IT demonstrated better effects than IV (Figure 6).

#### 3.4.5 Publication bias

**FIGURE 7:**
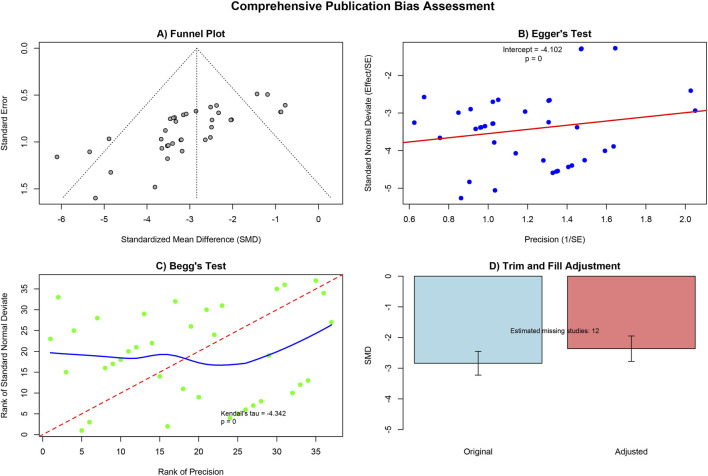
Results of publication bias detection. **(A)** Funnel plot; **(B)** Egger’s test; **(C)** Begg’s test; **(D)** Trim and fill adjustment.

We assessed publication bias for the most commonly reported outcome measure, MLI (n = 33). Funnel plots showed visual asymmetry (Figure 7A). Further Egger’s and Begg’s tests indicated the possibility of publication bias (Figures 7B,C). To address this bias, we performed a trim-and-fill analysis, estimating 12 potentially unpublished studies. After correction for publication bias, the pooled effect size remained statistically significant (SMD = −2.362, 95% CI: 2.776 to −1.948), indicating that despite publication bias, the conclusion that MSC significantly improves MLI is robust (Figure 7D; Supplementary Figure 1).

## 4 Discussion

This study provides a systematic quantitative synthesis of the effects of MSC transplantation in animal models simulating COPD pathology. Through a meta-analysis of 40 randomized controlled trials, we found that MSC transplantation significantly improved key pathophysiological features in animal models simulating COPD pathology, including the reduction of alveolar structural damage, modulation of inflammatory responses, and reduction of pulmonary cell apoptosis. Importantly, subgroup analyses revealed that both the transplantation route and dose were critical factors influencing therapeutic efficacy. These findings provide crucial evidence for the standardization of future preclinical research and the optimization of clinical translation strategies.

The typical pathological progression of COPD is characterized primarily by the development of emphysema. Our analysis indicates that MSC transplantation significantly reduces the mean linear intercept (MLI), suggesting its potential to alleviate alveolar structural damage. Current perspectives suggest that the core therapeutic mechanism of MSCs may primarily rely on their robust paracrine activity, which regulates the local microenvironment and promotes tissue repair and remodeling through the secretion of various bioactive factors, including hepatocyte growth factor (HGF), vascular endothelial growth factor (VEGF), and keratinocyte growth factor (KGF) (Yuan et al., 2025; Xiao et al., 2025; Khayatan et al., 2025). At the level of immune modulation, MSCs may help restore immune homeostasis by inducing macrophage polarization towards the anti-inflammatory M2 phenotype, inhibiting neutrophil infiltration, and regulating the balance of T cell subpopulations. Additionally, MSCs from various sources have been shown to possess anti-fibrotic potential and play a role in maintaining the integrity of pulmonary vascular endothelium, thereby supporting the function of the alveolar-capillary unit (Strizova et al., 2023; Broekman et al., 2016; Guarnier et al., 2023; Chen et al., 2022). These multiple mechanisms collectively provide a reasonable explanation for the observed improvement in alveolar structure (as indicated by the decrease in MLI).

Furthermore, our meta-analysis confirms that MSC transplantation is closely associated with the rebalancing of inflammation in animal models simulating COPD pathology, as evidenced by reduced levels of TNF-α and increased levels of IL-10. This finding supports the notion of MSCs having potent immunoregulatory functions. The underlying mechanisms may involve the promotion of macrophage polarization towards the M2 phenotype through the secretion of molecules such as prostaglandin E2 and TSG-6, thereby increasing the production of anti-inflammatory factors like IL-10 (Hezam et al., 2023; Liu et al., 2017). Additionally, MSCs may directly antagonize pro-inflammatory signaling pathways through the high expression of IL-1ra or inhibit core inflammatory pathways such as NF-κB by secreting specific miRNAs (Ortiz et al., 2007; Jeffries et al., 2019; Baranasic et al., 2023). *In vitro* studies also suggest that MSCs can downregulate the secretion of pro-inflammatory mediators from macrophages by inhibiting signaling pathways such as p38 MAPK (Gu et al., 2015).

Apoptosis is a crucial physiological process for maintaining lung tissue homeostasis, and its aberrant activation is considered one of the key mechanisms underlying COPD pathogenesis. Numerous studies have shown that excessive apoptosis of alveolar epithelial cells and pulmonary microvascular endothelial cells can lead to the destruction of lung parenchyma, rupture of alveolar septa, and enlargement of air spaces, ultimately resulting in irreversible structural changes characteristic of emphysema (Kennelly et al., 2016). Particularly under conditions of smoking or prolonged inflammatory stimuli, oxidative stress and inflammatory cytokines such as TNF-α and IL-1β can induce a caspase cascade through the activation of mitochondrial pathways, death receptor pathways, and endoplasmic reticulum stress pathways, leading to programmed cell death of lung cells (Fu et al., 2025; Wang T. et al., 2025). Our meta-analysis results indicate that MSC transplantation significantly reduces the number of TUNEL-positive cells, suggesting a stable and reproducible anti-apoptotic effect in animal models simulating COPD pathology. This finding is consistent with previous reports and further supports the hypothesis that MSCs confer lung protection by modulating apoptotic pathways. Specifically, MSCs can release various growth factors and cytokines, such as HGF, VEGF, and KGF, through paracrine mechanisms, activating downstream signaling pathways like PI3K/Akt and STAT3, which promote the expression of anti-apoptotic proteins (e.g., Bcl-2, Bcl-xL) and inhibit the activation of pro-apoptotic proteins (e.g., Bax, cleaved caspase-3) (Kennelly et al., 2016; Yew et al., 2011). The activation of the Akt signaling axis not only stabilizes mitochondrial membrane potential but also blocks the caspase-mediated cell death cascade, ultimately ensuring the survival of alveolar epithelial cells and microvascular endothelial cells (Zhang et al., 2025; Liu et al., 2025). Additionally, MSCs further alleviate cell damage induced by the inflammatory microenvironment by upregulating the anti-inflammatory factor IL-10 and downregulating the pro-inflammatory factor TNF-α, thereby indirectly inhibiting the apoptotic process.

Identifying the optimal transplantation strategy is crucial for advancing the successful treatment of COPD with MSCs (Guarnier et al., 2023). This study establishes, for the first time at an evidence-based level, that the route of administration and dosage are core determinants of the heterogeneity in MSC efficacy through systematic quantitative analysis. Subgroup analyses reveal that, at the same dosage, intratracheal transplantation outperforms intravenous administration in improving mean linear intercept (MLI), increasing IL-10 levels, and enhancing anti-apoptotic effects. The differences in efficacy may be attributed to the varying delivery efficiencies and pulmonary biodistribution of cells via different routes. Cells administered intravenously are prone to being sequestered or cleared by extra-pulmonary organs, whereas intratracheal transplantation allows for direct delivery to the target site, potentially enhancing local cellular bioavailability and prolonging their effects (Eggenhofer et al., 2012; Zheng et al., 2022; von Bahr et al., 2012; Lan et al., 2019; Nejaddehbashi et al., 2023; Antunes et al., 2014). Histological evidence indicates that MSC delivered via the intratracheal route predominantly localize in the peribronchial regions, where they repair damaged type II alveolar epithelial cells through paracrine mechanisms, improving alveolar structural collapse and ultimately establishing a functional regenerative microenvironment at the injury site. In contrast, cells infused intravenously primarily accumulate in the pulmonary capillaries or larger blood vessels (Lan et al., 2019; Zhang et al., 2014; Lan et al., 2017; Piatkowski et al., 2018). Cell survival rates and timing of delivery may also influence the therapeutic effects of MSCs; however, current research data do not provide insights into these aspects, indicating a need for future studies to focus more on the timing of MSC transplantation and their survival rates. Additionally, it is important to note that while some studies included in this meta-analysis utilized IP injection as an alternative route, our data show that its efficacy, particularly in enhancing the anti-inflammatory factor IL-10, is limited and inconsistent. This instability in efficacy, combined with the inherent pharmacokinetic disadvantages of the IP route for achieving targeted pulmonary delivery (such as first-pass effects and cell retention in the abdominal cavity), suggests that intraperitoneal injection is not the optimal strategy for MSC treatment in animal models simulating COPD pathology. Therefore, it is not recommended as a preferred choice for future standardized preclinical studies or clinical trials. Regarding the dose-response relationship, this study reveals a clear dose-dependent effect. There is a distinct positive dose-response characteristic in terms of alveolar structural remodeling and anti-apoptotic effects, with higher dosage groups (≥5 × 10^6^) generally exhibiting more pronounced therapeutic outcomes. However, the modulation of specific inflammatory markers (such as IL-10 and TNF-α) presents a complex nonlinear dose-response relationship, indicating that MSC interventions may have different dose thresholds for various pathological processes, particularly as the finely tuned immunomodulatory mechanisms are highly sensitive to fluctuations in cell numbers.

The findings of this study hold direct relevance for the design of future early clinical trials. First, regarding the route of administration, given the significant advantages of intratracheal transplantation observed in animal models, it is recommended that clinical trials prioritize exploring local delivery methods such as bronchoscopic administration or nebulized inhalation. Second, studies should be designed to include at least two dosage levels (for example, moderate and high doses based on body surface area calculations) to assess the dose-response relationship in humans. Finally, it is advisable to incorporate high-resolution CT quantitative metrics that reflect the degree of emphysema (such as lung density) as well as sensitive inflammatory biomarkers indicated by this meta-analysis (such as IL-10 and TNF-α) into the efficacy evaluation framework, in order to comprehensively validate the multiple mechanisms of action of MSCs.

In interpreting the aforementioned positive results, it is essential to carefully assess the potential for publication bias. The asymmetry observed in the funnel plot concerning the primary endpoint, MLI, along with the statistical test results, suggests the possibility that negative results may not have been published. However, the combined effect size, adjusted using the trim-and-fill method, remains highly significant, reinforcing the reliability of our core conclusion that MSCs effectively improve alveolar structure in animal models simulating COPD pathology. Nevertheless, interpretations of the findings from this study must take into account its inherent limitations, primarily including the heterogeneity of the animal models in terms of species, strain, and modeling methods, which may affect the generalizability of the results. Additionally, the majority of the original studies exhibited insufficient reporting on methodological key aspects such as random sequence generation and allocation concealment, which could introduce operational bias and impact the estimated efficacy. Most importantly, there is the issue of translational validity concerning the animal models included in this study. As previously mentioned, these models effectively simulate the structural emphysema and inflammatory state of human COPD, allowing us to evaluate the potential of MSCs to reverse these specific injuries. However, human COPD is a slowly progressive disease driven by years of environmental exposure (primarily smoking), accompanied by complex endogenous aging mechanisms and irreversible airway remodeling (Roth-Walter et al., 2024). In contrast, the models included in this study predominantly represent relatively stable pathological states formed after acute injury, and their microenvironments, disease drivers, and tissue self-repair capabilities may differ significantly from those of chronic human disease. Therefore, caution is warranted when extrapolating the significant efficacy of MSCs observed in these disease models to progressive human diseases. Future research should further validate the long-term efficacy of MSCs using models that more closely resemble the natural history of human COPD, such as long-term cigarette smoke exposure models, and explore their role in blocking or delaying disease progression. Additionally, our team may have limited direct experience in preclinical animal experimentation, which could potentially lead to bias in the evaluation and reporting of results. However, to minimize any methodological bias that may arise from this, all researchers involved in bias risk assessment and data extraction underwent specialized and rigorous training focused on the SYRCLE tool and the characteristics of animal experimental design. This standardized, guideline-based evaluation process effectively ensured the objectivity and rigor of the evidence integration in this study.

## 5 Conclusion

This study, through a meta-analysis of 40 randomized controlled animal experiments, found that MSCs significantly improve the alveolar structure in animal models simulating COPD pathology, reducing MLI, increasing levels of the anti-inflammatory factor IL-10, and suppressing the expression of the pro-inflammatory factor TNF-α as well as levels of apoptosis. Furthermore, intratracheal transplantation demonstrated superior efficacy compared to intravenous transplantation, and higher doses of MSCs exhibited more pronounced therapeutic effects. These findings provide important evidence for preclinical research and clinical translation in COPD, emphasizing the necessity of optimizing MSC transplantation strategies. However, it is important to note that the animal models included in this study were primarily based on acute injury, which mainly replicates the core pathological features of emphysema and inflammation seen in human COPD, rather than its typical chronic progressive course. The differences in disease progression between these models and human conditions are critical factors that need to be carefully considered in future clinical translation efforts.
