# Supplementary material for: Therapeutic effects of mesenchymal stromal cells transplantation in animal models of chronic obstructive pulmonary disease: a systematic review and meta-analysis of emphysema and lung inflammation models
Source: Front Cell Dev Biol. 2026 Jan 8;13:1739905. doi: 10.3389/fcell.2025.1739905 (PMC12823847; doi:10.3389/fcell.2025.1739905)
Supplement: Supplementary file 1 [file Table1.DOCX]

**Therapeutic Effects of Mesenchymal stromal cells Transplantation in Animal Models of Chronic Obstructive Pulmonary Disease: A Systematic Review and Meta-Analysis of Emphysema and Lung Inflammation Models**

**Table 1: Search strategies**

| **PubMed**  #1: "Stem Cells"[MeSH Terms]  #2: "stem cell"[Title/Abstract] OR "stem cells"[Title/Abstract] OR "mesenchymal stem cells"[Title/Abstract] OR "MSC"[Title/Abstract] OR "adipose-derived stem cells"[Title/Abstract] OR "ADSC"[Title/Abstract] OR "bone marrow stem cells"[Title/Abstract] OR "umbilical cord mesenchymal stem cells"[Title/Abstract] OR "hUC-MSC"[Title/Abstract]  #3: #1 AND #2  #4: "pulmonary disease, chronic obstructive"[MeSH Terms]  #5: "chronic obstructive pulmonary disease"[Title/Abstract] OR "COPD"[Title/Abstract]  #6: #4 or #5  #7: #3 and #6  **Web of science**  (TS=(“stem cell” OR “stem cells” OR “mesenchymal stem cells” OR “MSC” OR “adipose-derived stem cells” OR “ADSC” OR “bone marrow stem cells” OR “umbilical cord mesenchymal stem cells” OR “hUC-MSC”)) AND TS=(“chronic obstructive pulmonary disease” OR “COPD”)  **Embase**  #1: 'stem cell':ti,ab,kw OR 'stem cells':ti,ab,kw OR 'mesenchymal stem cells':ti,ab,kw OR 'msc':ti,ab,kw OR 'adipose-derived stem cells':ti,ab,kw OR 'adsc':ti,ab,kw OR 'bone marrow stem cells':ti,ab,kw OR 'umbilical cord mesenchymal stem cells':ti,ab,kw OR 'huc-msc':ti,ab,kw  #2: 'stem cell'/exp  #3: 'stem cells'/exp  #4: 'mesenchymal stem cells'/exp  #5: 'adipose-derived stem cells'/exp  #6: 'umbilical cord mesenchymal stem cells'/exp  #7: #1 OR #2 OR #3 OR #4 OR #5 OR #6  #8: 'chronic obstructive pulmonary disease':ti,ab,kw OR 'copd':ti,ab,kw  #9: 'chronic obstructive pulmonary disease'/exp  #10: #8 OR #9  #11: #7 AND #10  #12: #11 AND ('article'/it OR 'clinical trial'/it OR 'review'/it)  **Scopus**  TITLE-ABS-KEY ( "stem cell" OR "stem cells" OR "mesenchymal stem cells" OR "MSC" OR "adipose-derived stem cells" OR "ADSC" OR "bone marrow stem cells" OR "umbilical cord mesenchymal stem cells" OR "hUC-MSC" ) AND TITLE-ABS-KEY ( "chronic obstructive pulmonary disease" OR "COPD" ) AND ( LIMIT-TO ( DOCTYPE , "ar" ) OR LIMIT-TO ( DOCTYPE , "re" ) ) |
| --- |

**Table 2: Basic information of included studies**

| **No.** | **Author** | **Years** | **Country** |  | **Animal characteristics** | | | | | **Modeling method** | **MSC** | | **Transplantation route** | **Transplant dose** | **Control** |
| --- | --- | --- | --- | --- | --- | --- | --- | --- | --- | --- | --- | --- | --- | --- | --- |
|  |  |  |  | **Study type** | **Species** | **Gender** | **Weight** | **Age** | **sample size** |  | **Kinds** | **Source** |  |  |  |
| 1 | Nejaddehbashi | 2023 | Iran | RCT | SD rat | Male | / | / | 10/10 | Intratracheal injection of elastase | ADMSC | Gonadal fat pads of SD rats | intratracheal injection | 1×10^7 | PBS |
| 2 | Zhang | 2022 | China | RCT | BALB/C mice | Male | / | 8 weeks | 6/6 | Ozone exposure | ADMSC | Inguinal fat pads of BALB/C mice | intratracheal injection | 1×10^6 | PBS |
| 3 | Zhang | 2014 | China | RCT | SD rat | Female | 200-250g | / | 10/10 | Intratracheal instillation of lipopolysaccharide | BMSC | Femoral bone marrow of SD rats | intravenous injection | 4×10^6 | Blank control group |
| 4 | Zhen | 2010 | China | RCT | Lewis rat | / | / | / | 6/6 | Intratracheal instillation of papain | BMSC | Bone marrow of Lewis rats | intravenous injection | 4×10^6 | Blank control group |
| 5 | Gao | 2023 | China | RCT | SD rat | Male | / | 3-4 weeks | 16/16 | Cigarette smoking exposure | BMSC | Femoral bone marrow of SD rats | intravenous injection | 1×10^6 | Blank control group |
| 6 | Zhang | 2024 | China | RCT | SD rat | Male | 280-320g | 8 weeks | 5/5 | Intratracheal injection of elastase | hUMSC | Human umbilical cord | intravenous injection | 5×10^6 | Normal saline |
| 7 | Antunes | 2014 | Brazil | RCT | C57BL/6 mice | / | 20-25 g | 8 weeks | 7/7 | Intratracheal injection of elastase | BMSC | Bone marrow of C57BL/6 mice | intratracheal injection | 1×10^5 | Normal saline |
| 8 | Li | 2014 | China | RCT | SD rat | Female | 180-200 g | / | 15/15 | Cigarette smoke exposure and lipopolysaccharide injection | AFMSC | Amniotic fluid from pregnant SD rats | intratracheal injection | 4×10^6 | Blank control group |
| 9 | Wang | 2023 | China | RCT | C57BL/6 mice | Male | / | 6 weeks | 10/10 | Cigarette smoking exposure | BMSC | Bone marrow of C57BL/6 mice | intravenous injection | 1×10^6 | PBS |
| 10 | Poggio | 2018 | Brazil | RCT | C57BL/6 mice | Female | 20-25g | 8-10 weeks | 10/10 | Intratracheal injection of elastase | BMSC | Bone marrow of C57BL/6 mice | intratracheal injection | 1×10^5 | Normal saline |
| 11 | Lan | 2019 | China | RCT | ICR mice | Male | / | 8 weeks | 5/5 | Intratracheal injection of elastase | AFMSC | Amniotic fluid from pregnant ICR mice | intratracheal injection | 1×10^5 | PBS |
| 12 | Ridzuan | 2021 | Malaysia | RCT | SD rat | Male | 250-350 g | 8-9 weeks | 6/6 | Cigarette smoking exposure | hUMSC | Human umbilical cord | intratracheal injection | 2.5×10^6 | Blank control group |
| 13 | Park | 2018 | Korea | RCT | C57BL/6 mice | Female | / | / | 6/6 | Intratracheal injection of elastase | hUMSC | Human umbilical cord | intravenous injection | 1×10^4 | Blank control group |
| 14 | Schweitzer | 2011 | USA | RCT | C57BL/6 mice | Female | / | 12 weeks | 10/10 | Cigarette smoking exposure | ASMSC | Subcutaneous adipose tissue from humans | intravenous injection | 3×10^5 | Blank control group |
| 15 | Cho | 2017 | Korea | RCT | C57BL/6 mice | / | 20g | 6 weeks | 10/10 | Intratracheal injection of elastase | ASMSC | Subcutaneous adipose tissue from humans | intratracheal injection | 1×10^5 | Blank control group |
| 16 | Furuya | 2012 | Furuya | RCT | Wistar rat | Male | / | 8 weeks | 15/15 | Intratracheal injection of elastase | ASMSC | Dorsal fat pads of Wistar rats | intratracheal injection | 2.5×10^6 | Blank control group |
| 17 | Tibboel | 2014 | Canada | RCT | C57BL/6 mice | / | / | / | 30/25 | Intratracheal injection of elastase | BMSC | Femoral bone marrow of SD rats | intravenous injection | 5×10^5 | Blank control group |
| 18 | Kennelly | 2016 | Ireland | RCT | NSG mice |  | / | / | 5/5 | Intratracheal injection of elastase | BMSC | Human bone marrow | intravenous injection | 5×10^5 | PBS |
| 19 | Fujioka | 2021 | Japan | RCT | SCID beige mice | Female | / | 6 weeks | 10/10 | Intratracheal injection of elastase | ASMSC | Subcutaneous adipose tissue from humans | intravenous injection | 1×10^6 | PBS |
| 20 | Wu | 2025 | China | RCT | SD rat | / | 200-230 g | 8 weeks | 30/30 | Cigarette smoke exposure and lipopolysaccharide injection | BMSC | Bone marrow of SD rats | intravenous injection | 1×10^6 | PBS |
| 21 | Li | 2014 | China | RCT | SD rat | Male | 100-200g | / | 6/6 | Cigarette smoking exposure | BMSC | Human bone marrow | intravenous injection | 3×10^6 | PBS |
| 22 | Hong | 2016 | Korea | RCT | C57BL/6 mice | Female | / | 6 weeks | 10/10 | Intratracheal injection of elastase | ASMSC | Subcutaneous adipose tissue from humans | intravenous injection | 1×10^5 | PBS |
| 23 | Iwatake | 2024 | Japan | RCT | C57BL/6 mice | / | / | / | 10/10 | Intratracheal injection of elastase | hUMSC | Human umbilical cord | intravenous injection | 3×10^6 | Normal saline |
| 24 | Chen | 2021 | China | RCT | C57BL/6 mice | Male | 20-25g | 8 weeks | 10/10 | Cigarette smoking exposure | hUMSC | Human umbilical cord | intravenous injection | 3×10^6；1×10^7；3×10^7 | Normal saline |
| 25 | Huh | 2011 | Korea | RCT | Lewis rat | Female | / | 8 weeks | 10/10 | Cigarette smoking exposure | BMSC | Bone marrow of Lewis rats | intravenous injection | 6×10^6 | Serum-free DMEM |
| 26 | Laiman | 2022 | Indonesia | RCT | C57BL/6 mice | Male | / | 7 weeks | 10/10 | Intratracheal injection of elastase | hUMSC | Human umbilical cord | intravenous injection | 3×10^6;1×10^7 | PBS |
| 27 | Chen | 2022 | China | RCT | SD rat | / | / | / | 9/9 | VEGF receptor blocker SU5416 | hUMSC | Human umbilical cord | intravenous injection | 3×10^6 | PBS |
| 28 | Guan | 2012 | China | RCT | SD rat | Male | 224.2±10.8g | 8 weeks | 12/12 | Cigarette smoking exposure | BMSC | Bone marrow of SD rats | intratracheal injection | 6×10^6 | PBS |
| 29 | Li | 2017 | China | RCT | SD rat | Male | 170-200g | / | 8/8 | Cigarette smoking exposure | BMSC | Human bone marrow | intravenous injection | 3×10^6 | PBS |
| 30 | Gao | 2023 | China | RCT | C57BL/6 mice | Male | 18-20g | 8 weeks | 6/6/6 | Intratracheal injection of elastase | BMSC | Bone marrow of C57BL/6 mice | intratracheal injection | 5×10^5 | PBS |
| 31 | Zhen | 2008 | China | RCT | Lewis rat | Female | / | / | 10/10 | Intratracheal instillation of papain and cobalt-60 radiation | BMSC | Bone marrow of Lewis rats | intravenous injection | 4×10^6 | PBS |
| 32 | Cho | 2019 | South Korea | RCT | C57BL/6 mice | Male | / | / | 5/5 | Intratracheal injection of elastase | hUMSC | Human umbilical cord | intravenous injection | 5×10^4 | Normal saline |
| 33 | Song | 2014 | China | RCT | SD rat | Male | 250-280g | / | 5/5 | Cigarette smoking exposure | BMSC | Human bone marrow | intratracheal injection | 6×10^6 | PBS |
| 34 | Kim | 2014 | Korea | RCT | C57BL/6 mice | Female | 18-20g | 7 weeks | 6/6 | Intratracheal injection of elastase | MSC derived from human umbilical cord blood | Human umbilical cord blood | intravenous injection | 1×10^4; 2.5×10^4; 5×10^4; 1×10^5 | PBS |
| 35 | Peron | 2015 | Brazil | RCT | C57BL/6 mice | Female | / | 6-8 weeks | 5/5 | Cigarette smoking exposure | Human oviduct-derived MSCs | Human fallopian tubes | Peritoneal injection | 1×10^6 | PBS |
| 36 | Liu | 2015 | China | RCT | C57BL/6 mice | Female | / | 6 weeks | 6/6 | Cigarette smoking exposure | BMSC | Bone marrow of C57BL/6 mice | intravenous injection | 4×10^6 | PBS |
| 37 | Liu | 2017 | China | RCT | C57BL/6 mice | Female | / | 6 weeks | 6/6 | Cigarette smoking exposure and Haemophilus influenzae | BMSC | Bone marrow of C57BL/6 mice | intravenous injection | 4×10^6 | PBS |
| 38 | Fukui | 2019 | Japan | RCT | C57BL/6 mice | / | / | 8-10 weeks | 6/6 | Intratracheal injection of elastase | ASMSC | Adipose tissue from C57BL/6 mice | intravenous injection | 5×10^5 | Normal saline |
| 39 | TAKEDA | 2018 | USA | RCT | C57BL/6 mice | Female | / | 8-10 weeks | 8/8 | Intratracheal injection of elastase | BMSC | Bone marrow of C57BL/6 mice | intravenous injection | 1×10^6 | Normal saline |
| 40 | Gu | 2015 | China | RCT | SD rat | Male | 350±10g | 6-8 weeks | 8/8 | Cigarette smoking exposure and Haemophilus influenzae | BMSC | Femoral bone marrow of SD rats | intratracheal injection | 6×10^6 | Serum-free DMEM |
